# Ad‐E6/7‐HR vaccine improves the prophylactic and therapeutic efficacy in HPV‐associated cancers

**DOI:** 10.1002/ctm2.70305

**Published:** 2025-04-23

**Authors:** Yu Zhang, Ke Qiu, Jiayuan Ai, Maosen Xu, Binhan Wang, Aqu Alu, Chunjun Ye, Xiya Huang, Yu Zhang, Yingqiong Zhou, Zhiruo Song, Jie Shi, Yishan Lu, Yuquan Wei, Jianjun Ren, Yu Zhao, Ping Cheng, Xiawei Wei

**Affiliations:** ^1^ Department of Biotherapy Cancer Center and State Key Laboratory of Biotherapy West China Hospital Sichuan University Chengdu Sichuan China; ^2^ Department of Otolaryngology‐Head & Neck Surgery West China Hospital Sichuan University Chengdu Sichuan China

**Keywords:** adenovirus vaccine, cellular immunity, human‐papillomavirus‐associated cancers, oncology

## Abstract

**Background:**

High‐risk human papillomavirus (HPV), especially HPV16, is closely correlated with certain cancers. E6 and E7 proteins of HPV16 play critical roles in oncogenesis, making them optimal targets for treating HPV‐associated cancers. Here, we engineered an innovative vaccine, Ad‐E6/7‐HR, designed to evoke immune responses through the incorporation of self‐assembling heptad‐repeat 1 (HR1) and HR2 originated from Severe acute respiratory syndrome coronavirus 2.

**Methods:**

Ad‐E6/7‐HR was constructed utilising a replication‐defective human adenovirus serotype 5 vector and evaluated its immunogenicity and therapeutic efficacy in murine models. We verified the antitumour efficacy of the vaccine in TC‐1 subcutaneous and pulmonary models. Flow cytometry, enzyme‐linked immunospot assay, and immunofluorescence staining were used to assess the cellular immunogenicity of Ad‐E6/7‐HR.

**Results:**

Ad‐E6/7‐HR induced robust immune responses, significantly increasing antigen‐specific CD8^+^ T cells. The vaccine also enhanced memory T‐cell generation and induced potent cytokine secretion, as exemplified by interferon‐γ and tumour necrosis factor‐α. Ad‐E6/7‐HR conferred complete protection against tumour growth in the prophylactic model. In therapeutic settings, Ad‐E6/7‐HR significantly reduced tumour size and improved survival. Furthermore, Ad‐E6/7‐HR reshaped the tumour microenvironment by increased CD8^+^ T‐cell recruitment and reduced immunosuppressive cells, like myeloid‐derived suppressor cells and M2 macrophages, thereby enhancing antitumour immunity.

**Conclusions:**

By targeting HPV16 E6 and E7 proteins and leveraging the self‐assembling HR1 and HR2 sequences to enhance immune responses, Ad‐E6/7‐HR represented a promising candidate for preventing and treating HPV‐associated cancers. Further clinical investigation is warranted to evaluate its potential in human trials.

## Graphical abstract

Ad‐E6/7‐HR activates HPV16‐specific CD8^+^ T cells in the blood and spleen with enhanced IFN‐γ/TNF‐α secretion. Ad‐E6/7‐HR provides both preventative and therapeutic effects for HPV‐associated cancers. Ad‐E6/7‐HR reprograms TME via increased CD8^+^ T‐cell infiltration and reduced immunosuppressive MDSCs and M2 macrophages.

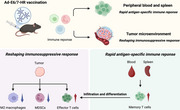

## 1 INTRODUCTION

Persistent infection with high‐risk human papillomavirus (HPV) can cause the occurrence of HPV‐associated cancers, including cervical cancer (CC), oropharyngeal squamous cell carcinoma (OPSCC), anal, vulvar, penile, as well as vaginal cancers.^1^ According to statistics, HPV is responsible for 90% of CC cases and 70% of OPSCC cases, with HPV16 being the predominant subtype.^2^, ^3^, ^4^ E6 and E7 proteins, encoded by high‐risk HPVs, are widely considered as oncoproteins that are closely linked to initiating HPV‐associated malignancies. By inhibiting the activity of p53 and pRb respectively. The two proteins contribute to uncontrolled cell proliferation, making them ideal targets for therapeutic interventions.^5^, ^6^, ^7^, ^8^ Although prophylactic vaccines, such as Gardasil‐9 and Cervarix, have been adopted and decreased the incidence of HPV‐associated cancers to a large extent,^9^, ^10^ they can't eliminate pre‐existing infections.^11^ Furthermore, various therapeutic vaccine modalities have undergone pre‐clinical and clinical trials, none of them have been officially approved yet.^12^, ^13^ Therefore, the development of innovative therapeutic strategies remains crucial in attenuating the burden of HPV‐associated cancers.

Virus‐like particles (VLPs) can be constructed by self‐assembling capability, mimicking native viruses in size and morphology.^14^ Due to the absence of genomic material, VLPs cannot replicate within hosts, ensuring a high safety profile.^15^ The heptad‐repeat (HR) 1 and 2 sequences, located in the S2 subunit of Severe acute respiratory syndrome coronavirus 2 (SARS‐CoV‐2), are highly conserved and exhibit self‐assembly capabilities that can form a 6‐helix bundle crucial for viral access.^16^ HR1 and HR2, belonging to VLPs, are emerging targets for fusion inhibitors and vaccines, with inhibitors demonstrating efficacy against SARS‐CoV‐2.^17^, ^18^, ^19^ Our team has shown that self‐assembly characteristic of HR1 and HR2 facilitates receptor‐binding domain trimerisation, enhancing vaccines’ immunogenicity and offering defence against emerging SARS‐CoV‐2 variants.^20^ Based on these insights, we propose a vaccine combining HPV16 E6/E7 with HR1/HR2 as fusion antigens, hypothesising that their trimeric structure will potentiate immune responses.

Viruses have the capability to infect cells, convey genetic instructions, and allow the expression of certain proteins, which enable them to become ideal vectors carrying specific antigens.^21^ Adenovirus (Ad) was employed as a vector delivering specific genes at first. To date, it has been regarded as vaccine vectors owing to the discovery of its immunogenic characteristic.^22^, ^23^ Ad has many compelling advantages as a vaccine vector. Firstly, it can infect various host cells, which is an outstanding feature as a vector. Secondly, its structural properties allow for the easy insertion of certain genes, facilitating the sufficient expression of designated genes or antigens. Thirdly, the transient nature of transfection and episomal expression significantly minimises the risk of genomic integration into host cells, thus making Ad a safer and more trustworthy vector.^24^ It's worth mentioning that the deletion of E1 and E3 regions enhances the safety and improves the transgene capability of Ad.^25^ Human adenovirus serotype 5 (Ad5) is the most extensively utilised adenovirus vector, underpinning the development of a range of vaccines targeting maladies incorporating Ebola virus infection,^26^ respiratory syncytial virus infection,^27^ SARS‐CoV‐2 infection,^28^ together with therapeutic interventions for colorectal cancer,^29^ metastatic castration‐resistant prostate cancer,^30^ and renal cell carcinoma.^31^ The safety and efficacy of Ad5‐based vaccines have been rigorously demonstrated through systemic trials. Despite the fact that several Ad‐based vaccines have been designed to treat HPV‐associated cancers, the efficacy of these vaccines is not as satisfactory as expected,^32^, ^33^ which highlights the urgent need for continued optimisation of Ad‐based vaccines to maximise their therapeutic potential.

Here, we designed a novel vaccine, Ad‐E6/7‐HR, incorporating sequences of HPV16 E6/E7, as well as HR1/HR2, based on the replication‐defective Ad5 to treat HPV‐associated cancers. We carried out detailed analyses of immunity elicited by Ad‐E6/7‐HR, along with its antitumour efficacy. Our results proved that single administration of Ad‐E6/7‐HR could evoke robust cellular immune responses, attenuate tumour progression, and extend survival, offering compelling support for the clinical management of HPV‐associated cancers.

## 2 RESULTS

### 2.1 Construction of Ad‐E6/7‐HR vaccine

**FIGURE 1:**
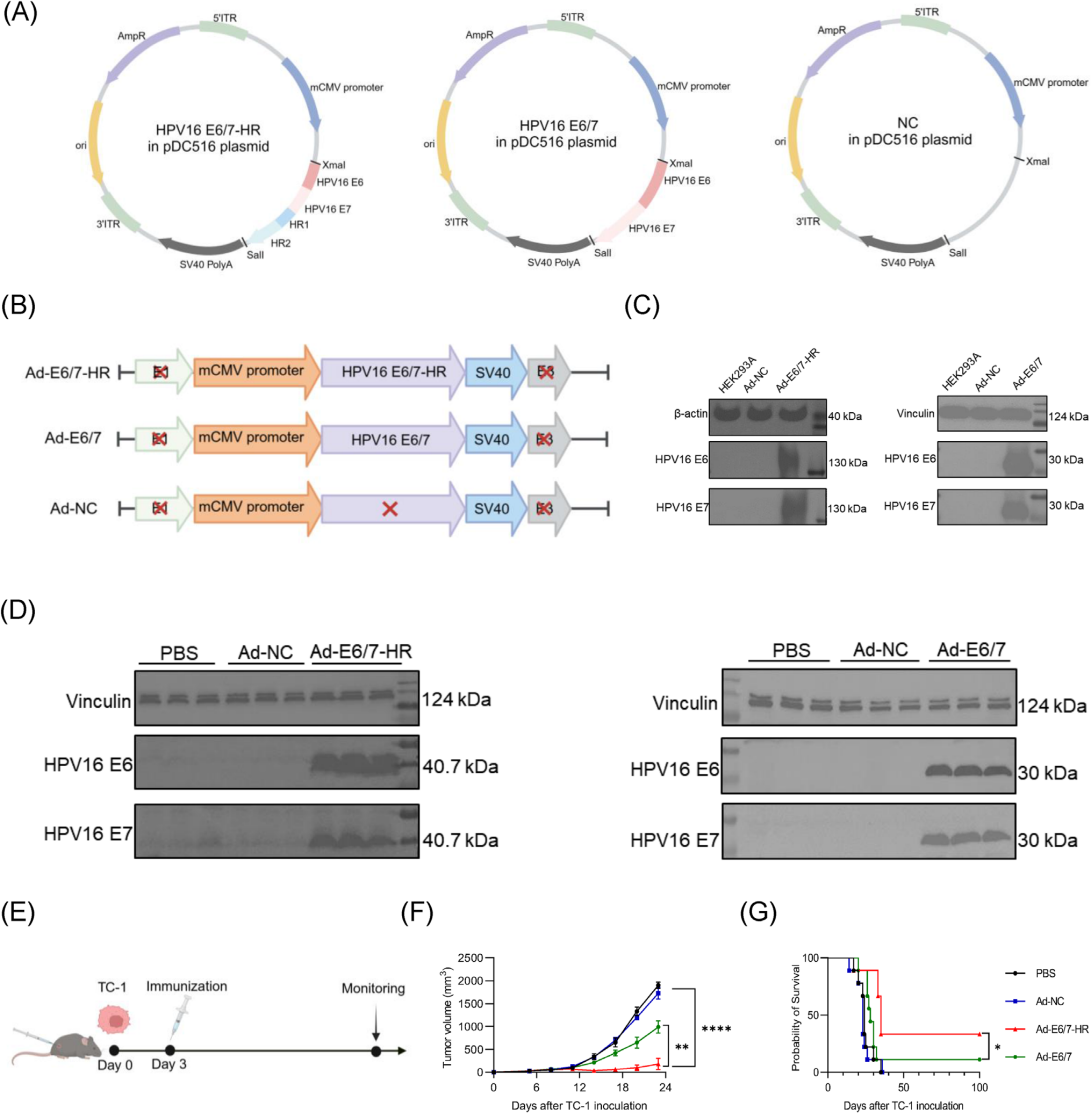
Design of Ad‐E6/7‐HR vaccine. (A, B) The schematic design of the vaccine. (C) Western blot analysis of expression of HPV16 E6 and E7 in HEK293A cells after transduced with Ad‐E6/7‐HR, Ad‐E6/7, or Ad‐NC. (D) Western blot analysis of expression of HPV16 E6 and E7 in vivo after mice were immunised with PBS, Ad‐NC, Ad‐E6/7‐HR, or Ad‐E6/7. (E) The timeline of the animal experiment. (F, G) Kinetics of tumour growth (F) and overall survival (G) were shown in a therapeutic model. (F), *n* = 6 per group. (G), *n* = 9 per group. Data are shown as mean ± SEM and analysed by two‐way ANOVA (F) and Log‐rank test (G). ^*^ *p* < .05, ^**^ *p* < .01, ^****^ *p* < .0001.

We employed a replication‐defective Ad5 vector to construct a novel vaccine named Ad‐E6/7‐HR, incorporating HPV16 E6 and E7, along with HR1 and HR2, arranged in tandem (Figure 1A and B). Ad‐E6/7, based on the same Ad5 vector (Figure 1A and B), was designed concurrently as control to evaluate the superiority of the Ad‐E6/7‐HR. Ad‐NC, a recombinant adenovirus vector devoid of target genes, serves as a negative control to exclude any immune responses that might arise from the vector itself (Figure 1A and B), rather than from fusion antigens. Ad‐NC, Ad‐E6/7, and Ad‐E6/7‐HR were then transduced into the HEK293A cells to detect the fusion protein expression. We observed protein expression in cells transduced with Ad‐E6/7‐HR and Ad‐E6/7 analysed by Western blot. Notably, fusion proteins were expressed as trimers attributable to the self‐assembly properties of HR1 and HR2, in line with previous studies (Figure 1C).^34^ Moreover, muscle tissues from mice injected with PBS, Ad‐NC, Ad‐E6/7‐HR, or Ad‐E6/7 were isolated and analysed 48 h post‐injection, demonstrating protein expression in vivo in Ad‐E6/7‐HR and Ad‐E6/7 groups (Figure 1D). We proposed a hypothesis that the trimeric form of E6 and E7 could enhance the immunogenicity and antitumour efficacy of Ad‐E6/7‐HR. Although Ad‐E6/7‐HR and Ad‐E6/7 didn't vary significantly in the antitumour efficacy in the prophylactic model (Figure S1A‐C), Ad‐E6/7‐HR exhibited superior efficacy in the therapeutic model (Figure 1E‐G), which suggested that Ad‐E6/7‐HR vaccine owned superior antitumour efficacy over conventional vaccine formulations, highlighting its prospect as a promising candidate for malignancies associated with HPV.

### 2.2 Trimeric structure enhances the immunogenicity of Ad‐E6/7‐HR

**FIGURE 2:**
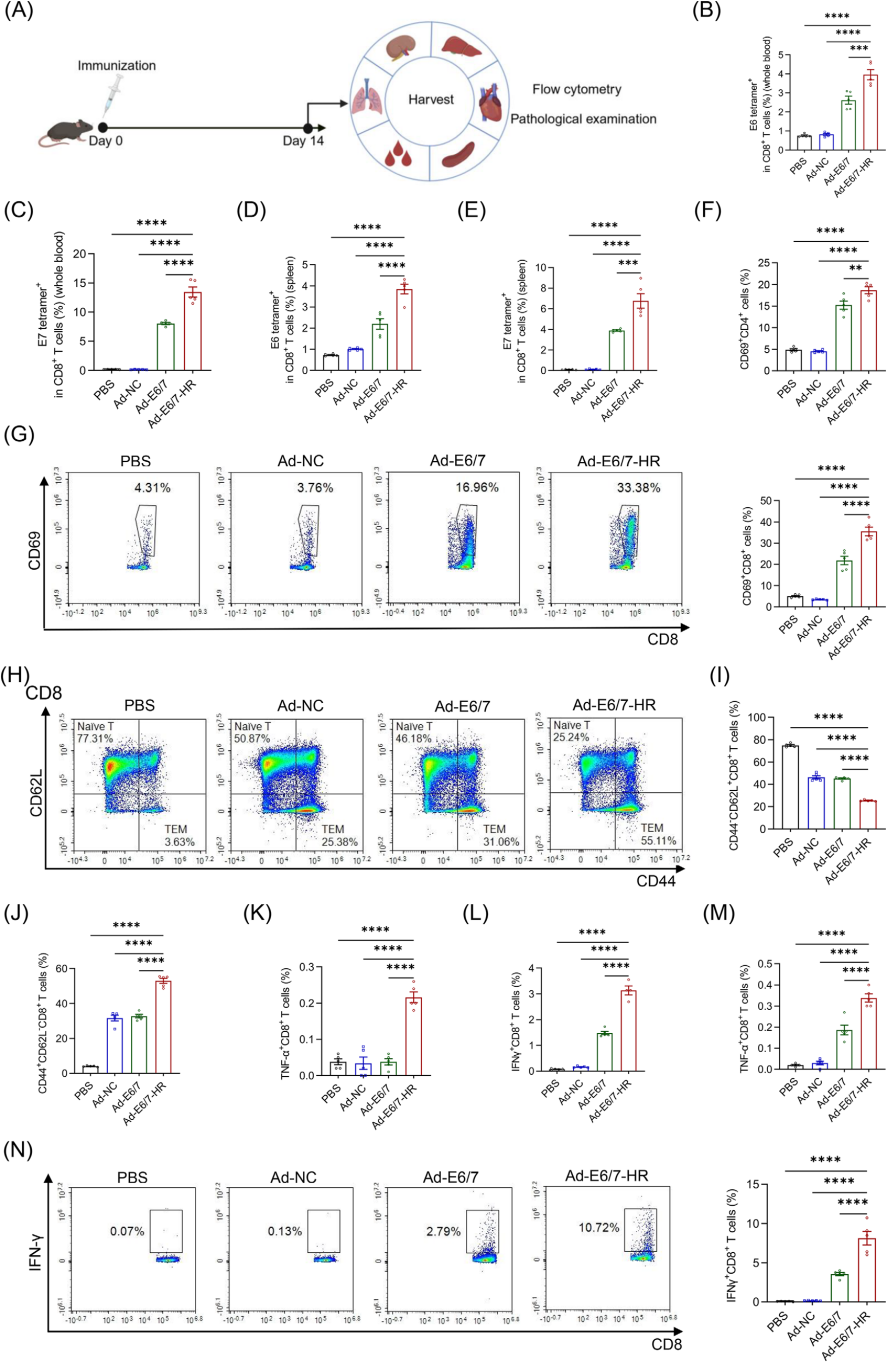
Ad‐E6/7‐HR vaccine can induce robust immune responses. (A) Strategy of the experiment. Mice were immunised intramuscularly with 5×10^9^ VP of Ad‐E6/7‐HR, Ad‐E6/7, Ad‐NC, or PBS respectively. (B, C) Percentages of E6 tetramer^+^ (B) and E7 tetramer^+^ (C) CD8^+^ T cells from blood. (D, E) Percentages of E6 tetramer^+^ (D) and E7 tetramer^+^ (E) CD8^+^ T cells in splenocytes. (F) Proportions of activated CD4^+^ T cells in splenocytes. (G) Representative plot and proportion of activated CD8^+^ T cells from spleen. (H) Representative plots of CD8^+^ naïve T cells (CD44^−^ CD62L^+^) and CD8^+^ effector memory T cells (TEM, CD44^+^ CD62L^−^). (I, J) Proportions of CD8^+^ naïve T cells (I) and CD8^+^ TEM (J) from spleen. (K, L) Percentages of TNF‐α^+^ CD8^+^ (K) and IFN‐γ^+^ CD8^+^ (L) T cells stimulated by E6 peptide pool. (M) Percentages of TNF‐α^+^ CD8^+^ T cells after stimulated by E7 peptide. (N) Representative plot and percentage of IFN‐γ^+^ CD8^+^ T cells after stimulated by E7 peptide. *n* = 5 per group. Data are shown as mean ± SEM and analysed by one‐way ANOVA with Tukey's multiple comparisons. ^*^ *p* < .05, ^**^ *p* < .01, ^***^ *p* < .001, ^****^ *p* < .0001.

To assess whether the trimeric configuration augmented the immunogenicity of Ad‐E6/7‐HR, mice were euthanised two weeks following a single intramuscular administration of Ad‐E6/7‐HR, Ad‐E6/7, Ad‐NC, or PBS (Figure 2A). Compared with the other three groups, Ad‐E6/7‐HR considerably increased the proportion of antigen‐specific CD8^+^ T cells in peripheral blood (Figure 2B and C). Further analysis of splenocytes revealed that Ad‐E6/7‐HR caused a pronounced increase in antigen‐specific CD8^+^ T cells, indicative of robust systemic immune responses conferred by the trimeric structure (Figure 2D and E). Activation of CD4^+^ and CD8^+^ T cells was boosted (Figure 2F and G), confirming that Ad‐E6/7‐HR vaccine effectively primed the adaptive immune system. Notably, we found a sharp decrease in CD8^+^ naïve T cells (Figure 2I) along with a substantial expansion in CD8^+^ effector memory T cells (TEM, Figure 2J), pivotal for providing robust and timely protection when bodies were faced with the same attack.

To figure out the activity of splenic CD8^+^ T cells, we detected the cytokine secretion following stimulation with E6 peptide pool or E7 peptide respectively. Intracellular cytokine staining (ICS) manifested that after stimulated by E6 peptide pool, a 5.7‐fold expansion in the ratio of tumour necrosis factor‐α^+^ (TNF‐α) CD8^+^ T cells was induced by Ad‐E6/7‐HR compared with the other groups (Figure 2K). Similarly, while interferon‐γ^+^ (IFN‐γ) CD8^+^ T cells were nearly undetectable in PBS and Ad‐NC groups, Ad‐E6/7‐HR elicited a 2.1‐fold higher frequency of these cells relative to Ad‐E6/7 (Figure 2L). Meanwhile, multiple folds of CD8^+^ T cells secreting IFN‐γ and TNF‐α were fostered upon E7 peptide stimulation in the Ad‐E6/7‐HR group (Figure 2M and N). In conclusion, Ad‐E6/7‐HR not only evoked potent immediate immune responses but also generated a memory pool that could offer durable protection against HPV‐associated cancers, underscoring the immunogenic superiority of Ad‐E6/7‐HR attributable to its trimeric confirmation.

### 2.3 Ad‐E6/7‐HR exhibits prophylactic efficacy in TC‐1 models

**FIGURE 3:**
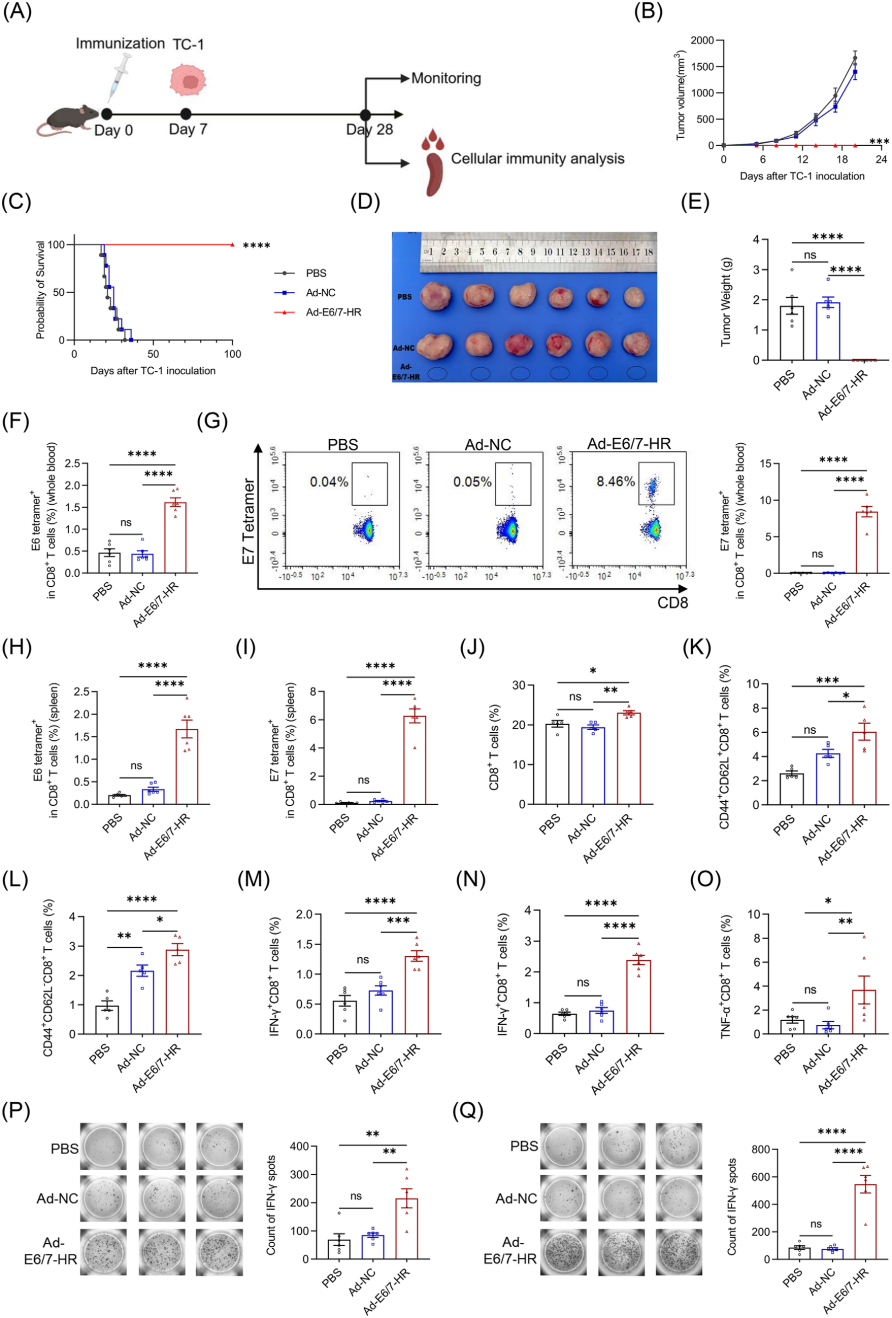
A single immunisation with Ad‐E6/7‐HR vaccine provides protection in a prophylactic TC‐1 model. (A) The timeline of experiments in the prophylactic model. (B–E) Kinetics of tumour growth (B) (*n* = 6 per group), overall survival (C) (*n* = 9 per group), representative images (D), and tumour weight of each group (E) were shown. (F) Percentages of E6 tetramer^+^ CD8^+^ T cells from blood. (G) Typical plot and proportion of E7 tetramer^+^ CD8^+^ T cells in blood. (H, I) Proportions of E6 tetramer^+^ (H) and E7 tetramer^+^ (I) CD8^+^ T cells in splenocytes. (J) Ratio of CD8^+^ T cells in the spleen. (K, L) Percentages of CD8^+^ central memory T cells (TCM, CD44^+^ CD62L^+^) (K) and CD8^+^ TEM (L) in the spleen. (M–O) Percentages of IFN‐γ‐producing and TNF‐α‐producing CD8^+^ T cells after stimulated by E6 peptide pool (M) or E7 peptide (N, O) separately. (P, Q) Images and quantitative plot of IFN‐γ analysed by ELISPOT stimulated by E6 peptide pool (P) or E7 peptide (Q). (E–Q) *n* = 6 per group. Data are shown as mean ± SEM and analysed by two‐way ANOVA (B), Log‐rank test (C), and one‐way ANOVA with Tukey's multiple comparisons (E–Q). ^*^ *p* < .05, ^**^ *p* < .01, ^***^ *p* < .001, ^****^ *p* < .0001, ns: not significant.

A prophylactic murine model was employed to detect the antitumour potential of Ad‐E6/7‐HR. A single dose of 5× 10^9^ viral particles (VP) of Ad‐E6/7‐HR, Ad‐NC, or PBS was administered intramuscularly to mice. Subsequent to immunisation, 2× 10^5^ TC‐1 cells were subcutaneously inoculated 7 days later (Figure 3A). Tumour growth was monitored. In contrast to other groups, all mice in the Ad‐E6/7‐HR group remained tumour‐free and survived throughout the study (Figure 3B and C). Tumour weights of the Ad‐E6/7‐HR group were substantially reduced relative to other groups (Figure 3D and E). Mice were sacrificed to investigate the cellular immune responses on Day 28. A pronounced increase in antigen‐specific CD8^+^ T cells was demonstrated, both in peripheral blood and spleen (Figure 3F‐I). A modest rise in overall CD8^+^ T cells indicated early lymphocyte activation (Figure 3J).^35^ Additionally, Ad‐E6/7‐HR induced the elevated generation of CD8^+^ central memory T cells (TCM) and CD8^+^ TEM subsets (Figure 3K and L), which could provide long‐lasting protection by swiftly generating activated cells, secreting cytokines, and recalling immune responses upon antigen re‐exposure.^36^ Ratios of CD8^+^ T cells expressing cytokines were considerably enhanced post‐treatment (Figure 3M‐O), with a more than 3‐fold increase after E7 peptide stimulation (Figure 3N and O). Consistent with results of ICS, higher levels of IFN‐γ stimulated by E6 peptide pool (Figure 3P) or E7 peptide (Figure 3Q) were observed in the Ad‐E6/7‐HR group than the other groups in the enzyme‐linked immunospot (ELISPOT) analysis. In a word, Ad‐E6/7‐HR could elicit robust cellular immune responses and provide antitumour protection in response to HPV‐associated cancers.

### 2.4 Ad‐E6/7‐HR provides long‐lasting protection

**FIGURE 4:**
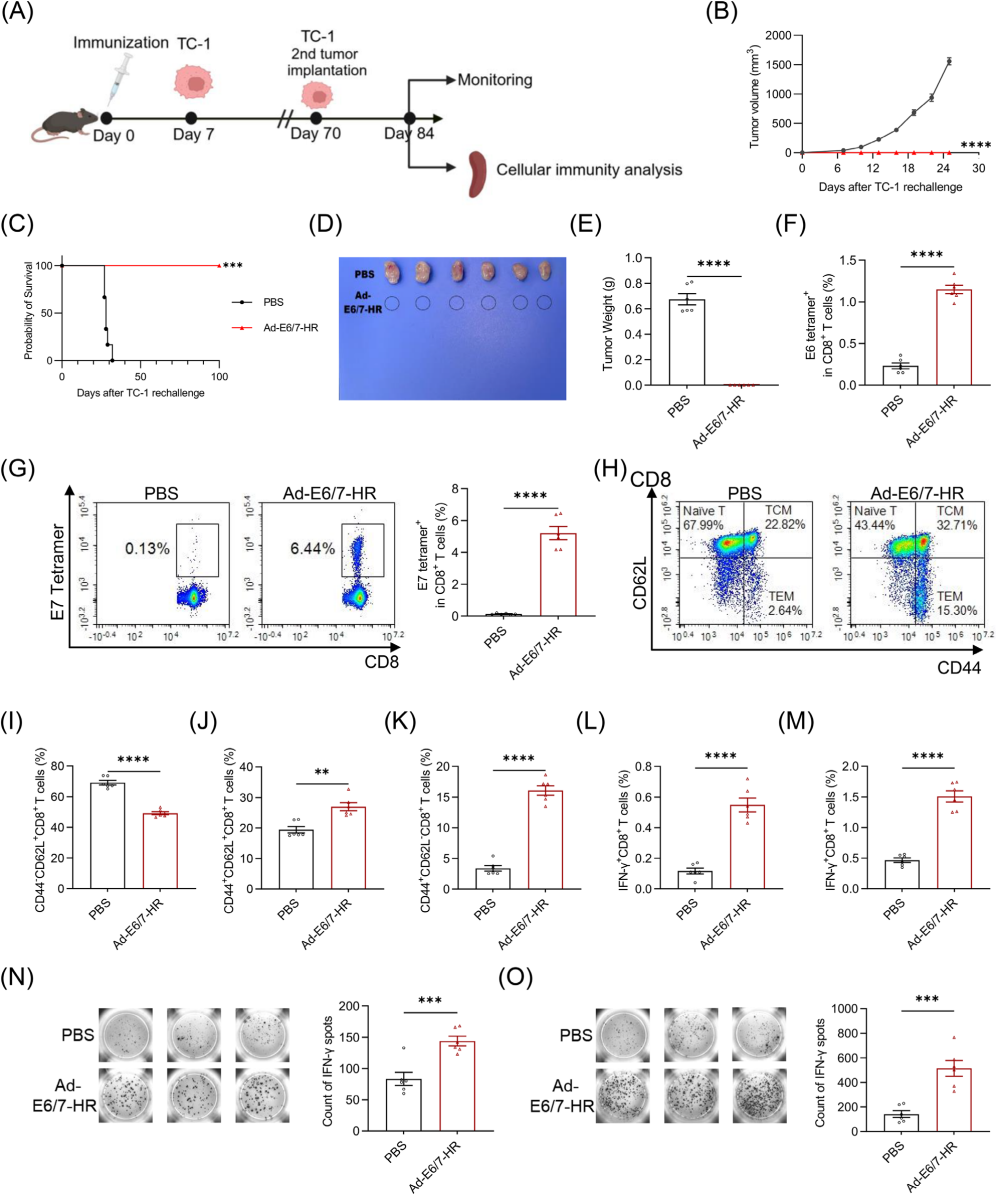
Ad‐E6/7‐HR vaccine provides long‐lasting protection from TC‐1 tumours. (A) Mice were inoculated with 2× 10^5^ TC‐1 cells 7 days after immunisation. Mice that survived were injected with 2× 10^5^ TC‐1 cells again on Day 70. Cellular immunity assays were conducted 14 days after rechallenge. (B‐E) Kinetics of tumour growth (B), overall survival (C), representative images (D), and tumour weight of each group (E) were shown. (F) Percentages of E6 tetramer^+^ CD8^+^ T cells in splenocytes. (G) Typical plot and proportion of E7 tetramer^+^ CD8^+^ T cells in splenocytes. (H) Representative plots of CD8^+^ naïve T, CD8^+^ TCM, and CD8^+^ TEM. (I–K) Proportions of CD8^+^ naïve T (I), CD8^+^ TCM (J), and CD8^+^ TEM (K) from spleen. (L, M) Percentages of IFN‐γ^+^ CD8^+^ T cells after stimulated by E6 peptide pool (L) or E7 peptide (M). (N, O) Images and quantitative plot of IFN‐γ analysed by ELISPOT stimulated by E6 peptide pool (N) or E7 peptide (O). *n* = 6 per group. Data are shown as mean ± SEM and analysed by two‐way ANOVA (B), Log‐rank test (C), and unpaired *t*‐test (E–G, I–O). ^**^ *p* < .01, ^***^ *p* < .001, ^****^ *p* < .0001.

To appraise the sustained antitumour protection provided by Ad‐E6/7‐HR, mice were rechallenged with 2× 10^5^ TC‐1 cells in the contralateral flank of dorsum on Day 70 (Figure 4A). Notably, Ad‐E6/7‐HR completely rescued mice from rechallenge and all mice remained tumour‐free (Figure 4B‐E), underscoring the enduring protective ability of the vaccine. Fourteen days post‐rechallenge, a subset of mice was euthanised, while the remaining mice were monitored till the end of the experiment. To elucidate the function of CD8^+^ T cells in the long‐lasting protection, immune responses in rechallenged mice were appraised. Immunised mice exhibited a nearly 4‐fold rise in E6 tetramer^+^ CD8^+^ T cells (Figure 4F), and a striking 35‐fold boost in E7 tetramer^+^ CD8^+^ T cells (Figure 4G). Ad‐E6/7‐HR evidently attenuated the ratio of CD8^+^ naïve T cells (Figure 4I), reflecting CD8^+^ T‐cell activation following tumour rechallenge. Concomitantly, CD8^+^ TCM and CD8^+^ TEM were substantially expanded, with an over 3‐fold increase in CD8^+^ TEM (Figure 4J and K), suggesting the establishment of a durable memory pool capable of immune surveillance. Moreover, stimulated by E6 peptide pool, the frequency of IFN‐γ^+^ CD8^+^ T cells was raised almost 5 times (Figure 4L), while E7 peptide stimulation resulted in a 3‐fold increase analysed by ICS (Figure 4M), which was further corroborated by ELISPOT assays (Figure 4N and O). In general, all these results implied that Ad‐E6/7‐HR could elicit compelling cellular immunity and offer long‐term protection against HPV‐associated malignancies.

### 2.5 Ad‐E6/7‐HR exhibits therapeutic efficacy in established tumours

**FIGURE 5:**
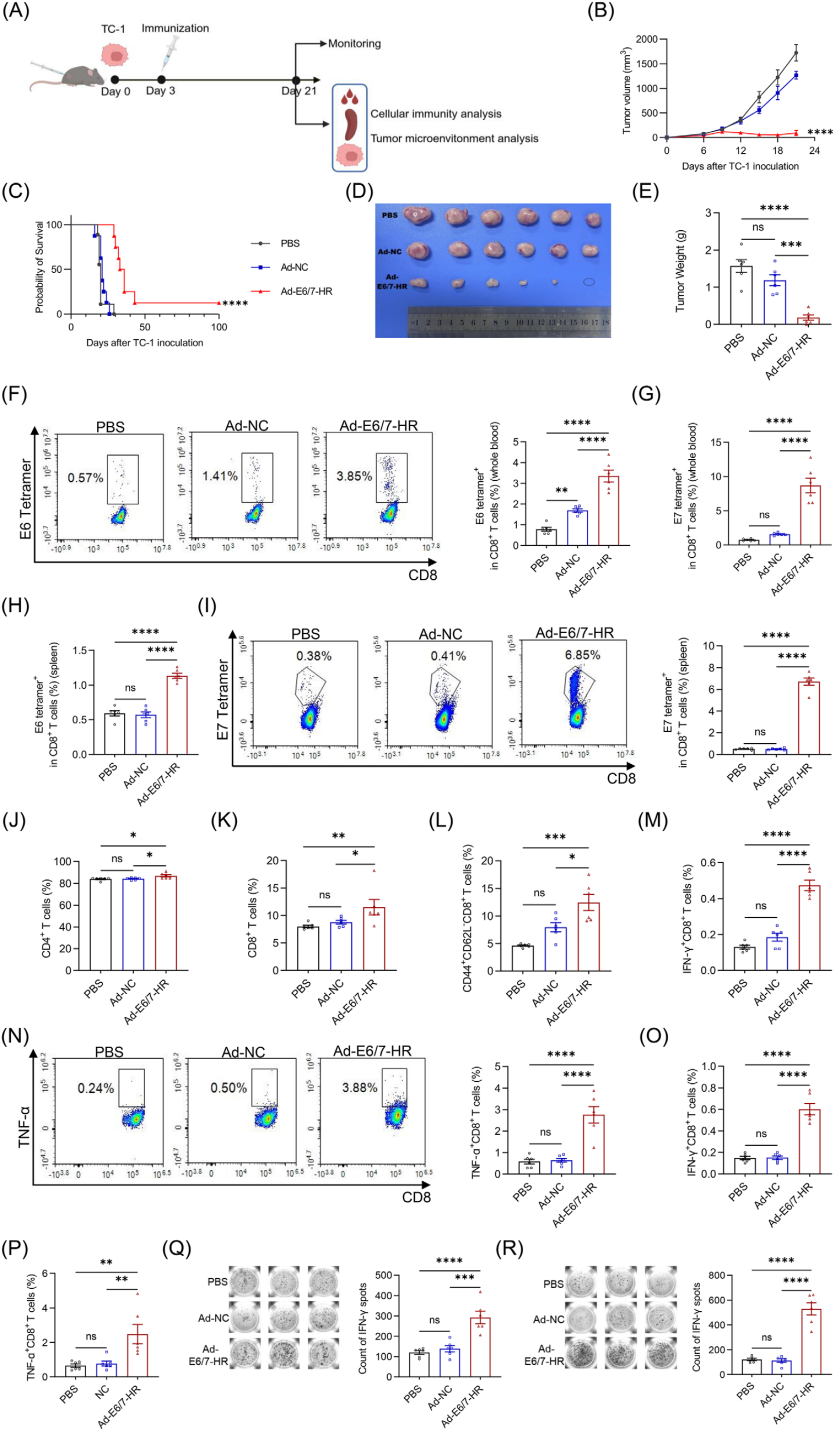
A single immunisation with Ad‐E6/7‐HR vaccine provides protection in a therapeutic TC‐1 model. (A) The timeline of experiments in the therapeutic model. (B–E) Kinetics of tumour growth (B) (*n* = 6 per group), overall survival (C) (*n* = 9 per group), representative images (D), and tumour weight of each group (E) were shown. (F) Typical plot and ratio of E6 tetramer^+^ CD8^+^ T cells from blood. (G) Percentages of E7 tetramer^+^ CD8^+^ T cells from blood. (H) Percentages of E6 tetramer^+^ CD8^+^ T cells from spleen. (I) Representative plot and ratio of E7 tetramer^+^ CD8^+^ T cells from spleen. (J, K) Frequencies of CD4^+^ (J) and CD8^+^ T cells (K). (L) Percentages of CD8^+^ TEM in the spleen. (M) Proportions of IFN‐γ^+^ CD8^+^ T cells stimulated by E6 peptide pool. (N) Symbolic plot and ratio of TNF‐α^+^ CD8^+^ T cells stimulated by E6 peptide pool. (O, P) Percentages of IFN‐γ^+^ CD8^+^ (O) and TNF‐α^+^ CD8^+^ (P) T cells stimulated by E7 peptide. (Q, R) Images and quantitative plot of IFN‐γ analysed by ELISPOT stimulated by E6 peptide pool (Q) or E7 peptide (R). (E–R) *n* = 6 per group. Data are shown as mean ± SEM and analysed by two‐way ANOVA (B), Log‐rank test (C), and one‐way ANOVA with Tukey's multiple comparisons (E–R). ^*^ *p* < .05, ^**^ *p* < .01, ^***^ *p* < .001, ^****^ *p* < .0001, ns: not significant.

We next evaluated the therapeutic efficacy of Ad‐E6/7‐HR in murine models. TC‐1 cells were subcutaneously inoculated and Ad‐E6/7‐HR, Ad‐NC, or PBS were intramuscularly injected 3 days later (Figure 5A). Tumour growth was markedly slowed in the Ad‐E6/7‐HR group, with smaller sizes of tumour and extended survival witnessed (Figure 5B‐E). To assess the immunological potential induced by Ad‐E6/7‐HR, peripheral blood and splenic cells were collected and analysed. Ratios of E6 tetramer^+^ CD8^+^ T cells in the Ad‐E6/7‐HR group was almost 3‐fold greater than those in the PBS group and nearly 2‐fold over those in the Ad‐NC group (Figure 5F). With respect to E7 tetramer^+^ CD8^+^ T cells, the proportion was 10 times over that of the PBS group and 5 times compared with the Ad‐NC group after treatment (Figure 5G). In accordance with what we found in the blood, levels of E6 tetramer^+^ CD8^+^ T cells were approximately twice higher than those in PBS and Ad‐NC groups (Figure 5H), whilst the ratio of E7 tetramer^+^ CD8^+^ T cells was almost 10‐fold larger (Figure 5I). Despite the minor rise of CD4^+^ T and CD8^+^ T cells (Figure 5J and K), Ad‐E6/7‐HR led to a notable expansion of CD8^+^ TEM in the spleen (Figure 5L). To evaluate the antigen specificity and functional capacity of T cells, splenic lymphocytes were isolated and restimulated with E6 peptide pool or E7 peptide respectively. As anticipated, multiple folds of IFN‐γ‐ and TNF‐α‐expressing CD8^+^ T cells were observed, correlated with the antitumour efficacy exertion (Figure 5M‐P). The ELISPOT assay further confirmed the IFN‐γ secretion, whose result was consistent with that of ICS (Figure 5Q and R). These findings highlighted the therapeutic potential of the Ad‐E6/7‐HR in halting tumour progression.

### 2.6 Ad‐E6/7‐HR modulates the tumour microenvironment

**FIGURE 6:**
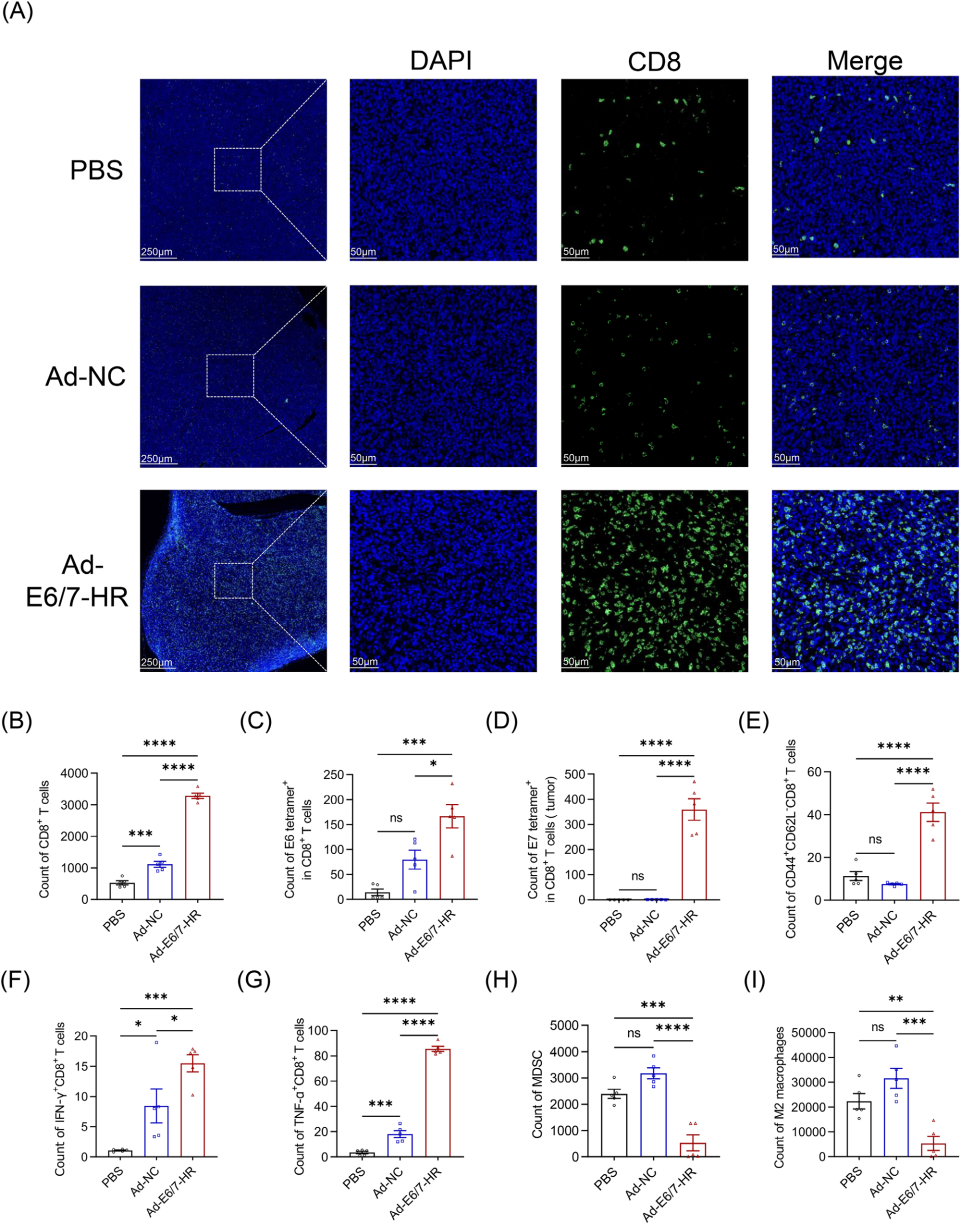
The alterations in the tumour microenvironment. (A) Images of immunofluorescence stained with anti‐CD8 antibodies in the tumour. Scale bar: 250 and 50 µm. (B) Absolute count of CD8^+^ T cells in the tumour. (C, D) Count of E6 tetramer^+^ (C) and E7 tetramer^+^ (D) CD8^+^ T cells in the tumour. (E) Count of CD8^+^ TEM in the tumour. (F, G) Count of IFN‐γ^+^ CD8^+^ (F) and TNF‐α^+^ CD8^+^ (G) T cells. (H) Count of MDSC in the tumour. (I) Count of M2 macrophages in the tumour. (B–I) *n* = 5 per group. Data are shown as mean ± SEM and analysed by one‐way ANOVA with Tukey's multiple comparisons (B–I). ^*^ *p* < .05, ^**^ *p* < .01, ^***^ *p* < .001, ^****^ *p* < .0001, ns: not significant.

To elucidate the mechanisms underlying tumour growth inhibition mediated by Ad‐E6/7‐HR, we extracted tumour tissues and explored cell infiltration in the tumour microenvironment (TME). The TME revealed a conspicuous rise of total CD8^+^ T‐cell infiltration as judged by immunofluorescence staining and flow cytometry (Figure 6A and B), implying that T‐cell accumulation probably mediated the effectiveness. By tetramer staining, we investigated that tetramer^+^ CD8^+^ T cells were dramatically increased (Figure 6C and D). Mice immunised with Ad‐E6/7‐HR generated a 10‐fold and 2‐fold expansion in E6 tetramer^+^ CD8^+^ T cells relative to PBS and Ad‐NC groups. Meanwhile, E7 tetramer^+^ CD8^+^ T cells were elevated by 1000‐fold and 390‐fold in the Ad‐E6/7‐HR group compared with PBS and Ad‐NC groups, respectively. Moreover, CD8^+^ TEM in the Ad‐E6/7‐HR group was almost 4‐fold greater than that in the other groups (Figure 6E), suggesting enhanced immune surveillance and long‐lasting protection. Corresponding with the efficacy of suppressing tumour growth, multiple times of functional CD8^+^ T cells were demonstrated (Figure 6F and G), highlighting the induction of robust antitumour immunity. Myeloid‐derived suppressor cells (MDSC) and M2 macrophages, related to immune suppression,^37^, ^38^ were considerably decreased in the Ad‐E6/7‐HR group (Figure 6H and I). To conclude, Ad‐E6/7‐HR contributed to the inhibition of tumour progression by inducing cellular immune responses and reshaping the TME conducive to tumour control.

## 3 DISCUSSION

Nowadays, surgery, radiotherapy, chemotherapy, immune checkpoint blockade therapy, and monoclonal antibodies are universally applied clinically to treat HPV‐associated cancers.^39^, ^40^ In spite of the positive results these therapies have achieved, drug resistance, reoccurrence, and metastasis remain challenging.^41^ Therefore, the development of effective vaccines treating HPV‐associated cancers has become a critical focus due to the significant global burden of these malignancies, among which persistent infection of HPV16 accounts for the major cause.^42^, ^43^ In this study, we designed an innovative vaccine, Ad‐E6/7‐HR, using a replication‐defective adenovirus vector incorporating HPV16 E6 and E7 along with HR1 and HR2 derived from SARS‐CoV‐2. Our results validated that Ad‐E6/7‐HR elicited robust antitumour immunity via mediating potent cellular responses and reshaping the TME, making it a promising candidate for treating HPV‐associated cancers.

An indispensable concern in the development of the vaccine is the potential oncogenic risk posed by the inclusion of oncogenic antigens. To address this concern, we engineered a fusion antigen by combining E6 and E7 sequences into a single open reading frame. Meanwhile, we mutated cysteine at position 24 and glutamic acid at position 26 of HPV16 E7 into glycine, inactivating the binding sites to pRb, which has been experimentally confirmed to eliminate cellular transformation and immortalisation capability, possessing enhanced safety feature.^44^ HR1 and HR2, derived from non‐oncogenic SARS‐CoV‐2, are known to contribute to form a stable trimeric structure, which enhances the immunogenicity of the vaccine without compromising safety.^17^, ^19^ Furthermore, the replication‐deficient nature of the adenovirus vector mitigates risk of oncogenesis, ensuring crucial advantages for therapeutic application.

The introduction of HR1 and HR2 was strategically chosen based on the intrinsic capacity for trimeric self‐assembly, a structural feature demonstrated to confer effective protection against infection.^16^, ^20^ We hypothesised that by combining these sequences with HPV16 E6 and E7, the immunogenicity of the vaccine would be enhanced. By systemic investigations in vivo and in vitro, we found that the trimeric configuration of the vaccine could not only substantially augmented immunogenicity but also exhibited superior antitumour efficacy in TC‐1 tumour models.

Ad‐E6/7‐HR exhibited excellent antitumour efficacy in murine models, the prophylactic efficacy of Ad‐E6/7‐HR was underscored by complete tumour protection in a murine model, with immunised mice remaining tumour‐free throughout monitoring. The robust cellular immune responses and memory T‐cell subsets underpinned the effectiveness of Ad‐E6/7‐HR in eliciting systemic and long‐term immunity. In a therapeutic setting, slower progression and extended survival were displayed after treatment. As anticipated, antigen‐specific CD8^+^ T cells, accompanied by cytokine‐expressing CD8^+^ T cells were considerably expanded, implying the capability of the vaccine in reversing tumour progression in established tumours.

To further elucidate the mechanism of antitumour efficacy, we analysed the alteration of TME. On one hand, a marked rise in CD8^+^ T‐cell infiltration was noticed, correlated with powerful management of tumour development and favourable prognosis.^45^, ^46^ Ad‐E6/7‐HR led to greater number of functional CD8^+^ T cells compared with the others, which was consistent with published discoveries that CD8^+^ T cells are of critical significance in mediating immune responses in TC‐1 models.^47^, ^48^ On the other hand, we observed a pronounced decrease in MDSC and M2 macrophages, which are typically associated with immune suppression and poor prognosis. The dual effect collectively emphasised the ability of Ad‐E6/7‐HR to reprogram the TME into an immunostimulatory landscape, not only fostering antitumour immunity but also counteracting immune evasion.

Cisplatin‐based chemotherapy and immune checkpoint therapy like anti‐programmed death‐1 (PD‐1) therapy have been widely employed in cancer treatment. We investigated potential benefits of combining the Ad‐E6/7‐HR vaccine with established treatment modalities. The combined therapy demonstrated superior tumour control and prolonged survival, outperforming anti‐PD‐1 monotherapy (Figure S4A‐C). Meanwhile, we also evaluated the potential synergistic effects of combining Ad‐E6/7‐HR with cisplatin and paclitaxel (Figure S4D). The combination significantly extended survival compared with chemotherapy alone (Figure S4E and F). These findings underscored the versatility of Ad‐E6/7‐HR as a therapeutic agent to achieve improved clinical outcomes, holding promise for addressing limitations of monotherapy, such as immune evasion and drug resistance, in the treatment of HPV‐associated cancers.

Research has confirmed that HPV‐associated cancers are prone to distant metastases in organs including lung, liver, and bones.^49^, ^50^ Considering this, we employed a pulmonary metastasis model to further assess the efficacy of the vaccine. Strikingly, we noticed that Ad‐E6/7‐HR could completely reverse the tumour dissemination and inhibit metastasis in vivo in both therapeutic and prophylactic settings (Figure S5A‐F), paving the way for further investigation into the broader application of Ad‐E6/7‐HR, particularly in advanced HPV‐associated malignancies.

To assess the safety of the Ad‐E6/7‐HR vaccine, mice that received a single dose of Ad‐E6/7‐HR vaccine were monitored. No adverse events, as exemplified by fur loss and behavioural changes were detected. Body weight didn't alter substantially (Figure S6A). Notable variations were not seen in the major organs of mice (Figure S6B). In addition, serum biochemical parameters exhibited no dramatic abnormalities between the Ad‐E6/7‐HR group and the other groups, confirming that the vaccine showed no detrimental effects on mice (Figure S6C). Taken together, these results indicated the excellent safety profile of the Ad‐E6/7‐HR vaccine.

In comparison to commercial HPV vaccines, such as Gardasil‐9, which are on the basis of VLPs originated from L1 proteins and primarily elicit L1‐specific neutralising bodies to prevent infection,^51^ the Ad‐E6/7‐HR vaccine targets E6 and E7 proteins and confers protection mainly through specific cellular immunity, enabling it not only prevent infection but also treat HPV‐driven malignancies, positioning it a viable option for application in established cancers. Moreover, the vaccine acquires the antitumour capacity with a single dose regimen, overcoming the logistical challenges associated with the multi‐dose schedules required by current HPV vaccines, aligned with the recommendation proposed by the World Health Organization to simplify vaccination protocols to improve vaccine accessibility.^52^ The advancement of Ad‐E6/7‐HR to provide durable protection in a single dose administration bridges the gap between prevention and treatment of HPV‐associated cancers, signifying its potential as a transformative approach to a major global health challenge.

Despite the promising results, there are several limitations in this study. While the design of the vaccine is of highly safety, the potential oncogenicity of E6 protein remains unaddressed and warrants further optimisation.^53^ Besides, orthotopic tumour models ought to be adopted to better recapitulate clinical settings. Further investigation is required. Moreover, while the current findings originate from murine models, the clinical translation remains a crucial hurdle due to inherent differences in immune responses between mice and human, which desperately needs thorough research to mitigate the translational barrier.

## 4 MATERIALS AND METHODS

### 4.1 Design of the vaccine

Sequences including the sequences of HPV16 E6 and E7 along with HR1 (916‐966 aa) and HR2 (1157‐1203 aa) were cloned into shuttle plasmids pDC516. Site directed mutation was used to fuse the E6 and E7 proteins. Moreover, sequence of E7 contains two mutations (C24G and E26G) to modify the Rb binding sites of E7. HEK293A cells were cotransfected with shuttle plasmids pDC516 containing HPV16 E6/7‐HR or HPV16 E6/7 gene and backbone plasmid pBHGfrtdelE13FLP. Ad‐E6/7‐HR and Ad‐E6/7 were prepared through an array of procedures including amplification, concentration, purification, and characterisation of Ad vectors.

### 4.2 Mice

Vital River (Beijing, China) supplied female C57BL/6 mice (6‐8 weeks old). Mice were reared under specific‐pathogen‐free conditions.

### 4.3 Cell lines

HEK293A were purchased from ATCC. TC‐1 cells, established through genetic modification, incorporating HPV16 E6/E7 and c‐ras oncogenes, were bought from Xiamen Immocell Biotechnology Company. HEK293A and TC‐1 were cultured in Dulbecco's modified Eagle's medium (DMEM; Gibco) and RPMI 1640 (Gibco) with 10% fetal bovine serum (FBS; Corning), penicillin (100 U/mL), and streptomycin (100 mg/mL). All cells were cultured at 37°C and 5% carbon dioxide.

### 4.4 Tumour challenge and treatment experiments

For therapeutic tumour models, 2× 10^5^ TC‐1 cells were subcutaneously injected in the right flank of dorsum on Day 0, 5× 10^9^ VP vaccine was injected intramuscularly on the right leg of mice 3 days later, when the tumour was palpable but not measurable. For prophylactic model, on Day 0, 5× 10^9^ VP vaccine was injected intramuscularly, followed by subcutaneous implantation of 2× 10^5^ TC‐1 cells 7 days later. For tumour rechallenging model, 2× 10^5^ TC‐1 cells were injected subcutaneously 70 days post‐vaccination. Tumour sizes were measured every 3 days. Tumour volumes were calculated using the equation: volume (mm^3^) = width× width× length× 0.52. 21 days post‐tumour inoculation, mice were euthanised.

For therapeutic tumour models in the pulmonary metastasis model, on Day 0, 3× 10^5^ TC‐1 cells were injected intravenously, 5× 10^9^ VP vaccine was injected intramuscularly on the right leg of mice on the next day. For prophylactic model, 5× 10^9^ VP vaccine was injected intramuscularly on Day 0 and 3× 10^5^ TC‐1 cells were implanted intravenously 7 days later. Sixteen days post‐tumour implantation, mice were sacrificed.

### 4.5 Tissue preparation

Tumour tissues were digested by 1 mg/mL collagenase Type I as well as 0.5 mg/mL collagenase Type IV (all from Sigma‐Aldrich) in RPMI 1640 basic medium, and incubated for 1 h at 37 °C. Cell suspension was filtered through 70 µM strainer (Falcon) and lysed by red blood cell (RBC) lysis buffer. After washes, cells were maintained in phosphate‐buffered solution (PBS). Spleens were mechanically disrupted through a 70 µM strainer. Lymphocytes from spleens were isolated by density gradient centrifugation. Subsequent to RBC lysis and washes, cells were resuspended in PBS. Lymphocytes derived from whole blood were isolated in RBC lysis buffer for several times and resuspended in PBS.

### 4.6 Flow cytometry

Lymphocytes from tumours were incubated with antibodies (1:100 dilution, BioLegend) at 4°C for 30 min. For ICS, cells were stained for surface markers at first. Then, cells were fixed and permeabilised with BD cytofix/Cytoperm™ Fixation/Permeabilization Kit (BD Bioscience) and stained with antibodies for 1 h at room temperature.

To evaluate cellular immune responses, splenic lymphocytes were isolated and cultivated. E7‐derived peptide (2 µg/mL, RAHYNIVTF) and E6‐derived peptide pool (4 µg/mL) were added respectively to stimulate cells. Brefeldin A (BD Biosciences) was added to impede cytokine secretion 6 h before staining. Upon completion of surface marker staining, cells were fixed, permeabilised, and incubated with intracellular antibodies. For tetramer staining, cells from spleens were stained with PE‐conjugated E6‐Tetramer and PE‐conjugated E7‐Tetramer (all from BetterGen) respectively. Cells were stained at 4°C for 1 h.

Flow cytometry was performed and analysed by NovoExpress® software (1.3.0, ACEA Biosciences, Inc., San Diego, CA).

### 4.7 ELISPOT assay

Lymphocytes were harvested from spleens and seeded (1.5× 10^5^ cells per well) in nitrocellulose‐bottom plates (MABTECH) and incubated with E7‐derived peptide (2 µg/mL) and E6‐derived peptide pool (4 µg/mL) respectively for at least 12 h in vitro. The detection of secreted IFN‐γ was conducted following protocols. Spots were then quantified and analysed.

### 4.8 Western blot analysis

HEK293A cells were transduced with adenovirus for 48 h. Then cell lysis buffer (Beyotime Institute of Biotechnology) supplemented with proteinase inhibitor cocktail (Sigma‐Aldrich) were used to lyse samples. Moreover, muscle tissues at the injection site were isolated and extracted 48 h post‐vaccination for sample preparation. Proteins were separated by polyacrylamide gel and non‐denaturing electrophoresis separately, and transferred to polyvinylidene difluoride membranes. After blocked with 5% non‐fat milk, membranes were incubated with antibodies overnight at 4°C: rabbit anti‐HPV16‐E6 (GeneTex, 1:1000), rabbit anti‐HPV16‐E7 (GeneTex, 1:1000), mouse anti‐actin (HUABIO, 1:1000), and mouse anti‐vinculin (Sigma, 1:1000). Secondary antibody incubation was performed using horseradish peroxidase‐conjugated goat anti‐rabbit (Abcam, 1:5000) and goat anti‐mouse (Abcam, 1:10 000). Plots were shown by ChemiDoc Touch (Bio‐Rad).

### 4.9 Immunofluorescence staining

Sections of tumour tissues were used for immunofluorescence staining. After deparaffinisation, hydration via alcohol gradient, antigen retrieval, and blocking, rabbit anti‐CD8 antibody (Cell Signaling Technology, 1:250) was utilised to incubate sections at 4°C overnight. Sections were then exposed to Alexa Fluor™ 488‐labelled goat anti‐rabbit secondary antibody (Invitrogen, 1:1000) for 1 h at ambient temperature.

### 4.10 Statistical analysis

Statistics were analysed by GraphPad Prism 9.4.0 (GraphPad, San Diego, CA). One‐way analysis of variance, two‐way analysis of variance, Student–Newman–Keuls test, and log rank tests were used and data was shown as mean ± SEM. Statistical significance was determined at *p* < .05.

## AUTHOR CONTRIBUTIONS

Yuquan Wei, Jianjun Ren, Yu Zhao, Ping Cheng, and Xiawei Wei developed the project and offered main direction of this study. Ping Cheng performed the construction of vaccines. Yu Zhang, Jiayuan Ai, and Maosen Xu conducted immunisations to mice. Yu Zhang, Jiayuan Ai, Maosen Xu, Binhan Wang, Aqu Alu, Chunjun Ye, Xiya Huang, Yu Zhang, Yingqiong Zhou, Zhiruo Song, Jie Shi, and Yishan Lu collected lymphocytes from blood and spleens and performed flow cytometry. Yishan Lu collected serum for biochemical analysis. Yu Zhang and Jiayuan Ai performed the ELISPOT assay. Yu Zhang, Ke Qiu, Jiayuan Ai, and Maosen Xu participated in data analysis. Yu Zhang and Ke Qiu wrote the manuscript. All authors have read and agreed to the submitted version of the manuscript.

## CONFLICT OF INTEREST STATEMENT

The authors declare no conflict of interest.

## ETHICS STATEMENT

Sichuan University's Institutional Animal Care and Use Committee gave the approval for all animal studies.

## Supporting information



Supporting Information

Supporting Information

Supporting Information

Supporting Information

Supporting Information

Supporting Information
