# Supplementary figures and images for: Ad‐E6/7‐HR vaccine improves the prophylactic and therapeutic efficacy in HPV‐associated cancers
Source: Clin Transl Med. 2025 Apr 23;15(4):e70305. doi: 10.1002/ctm2.70305 (PMC12017896; doi:10.1002/ctm2.70305)

A

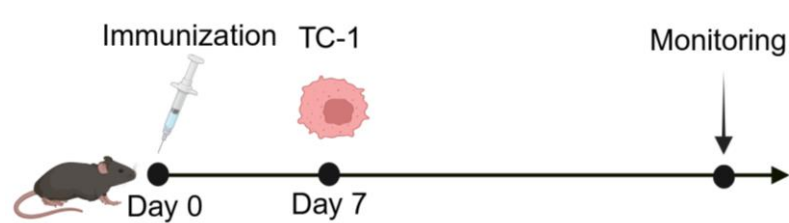

B

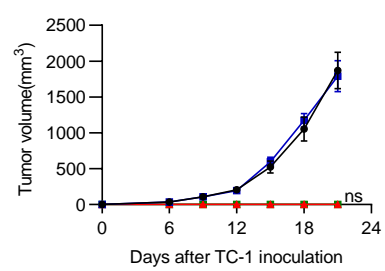

C

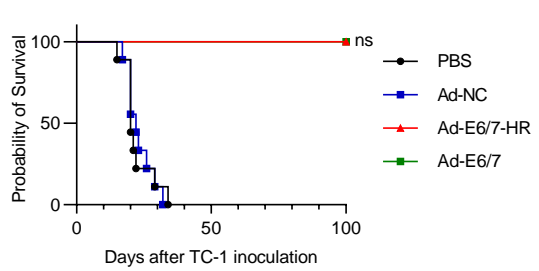

Supplement: Supplementary file 1 — Supporting Information [file CTM2-15-e70305-s006.pdf]

A

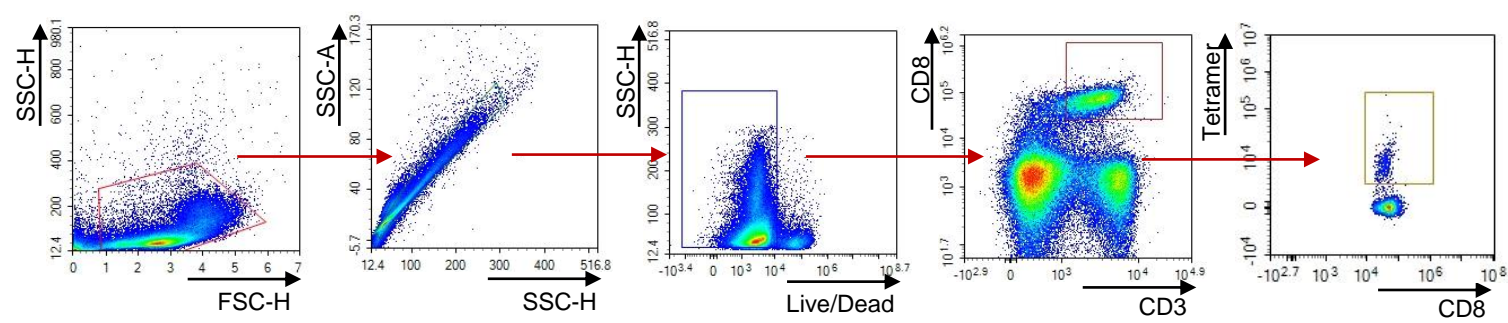

B

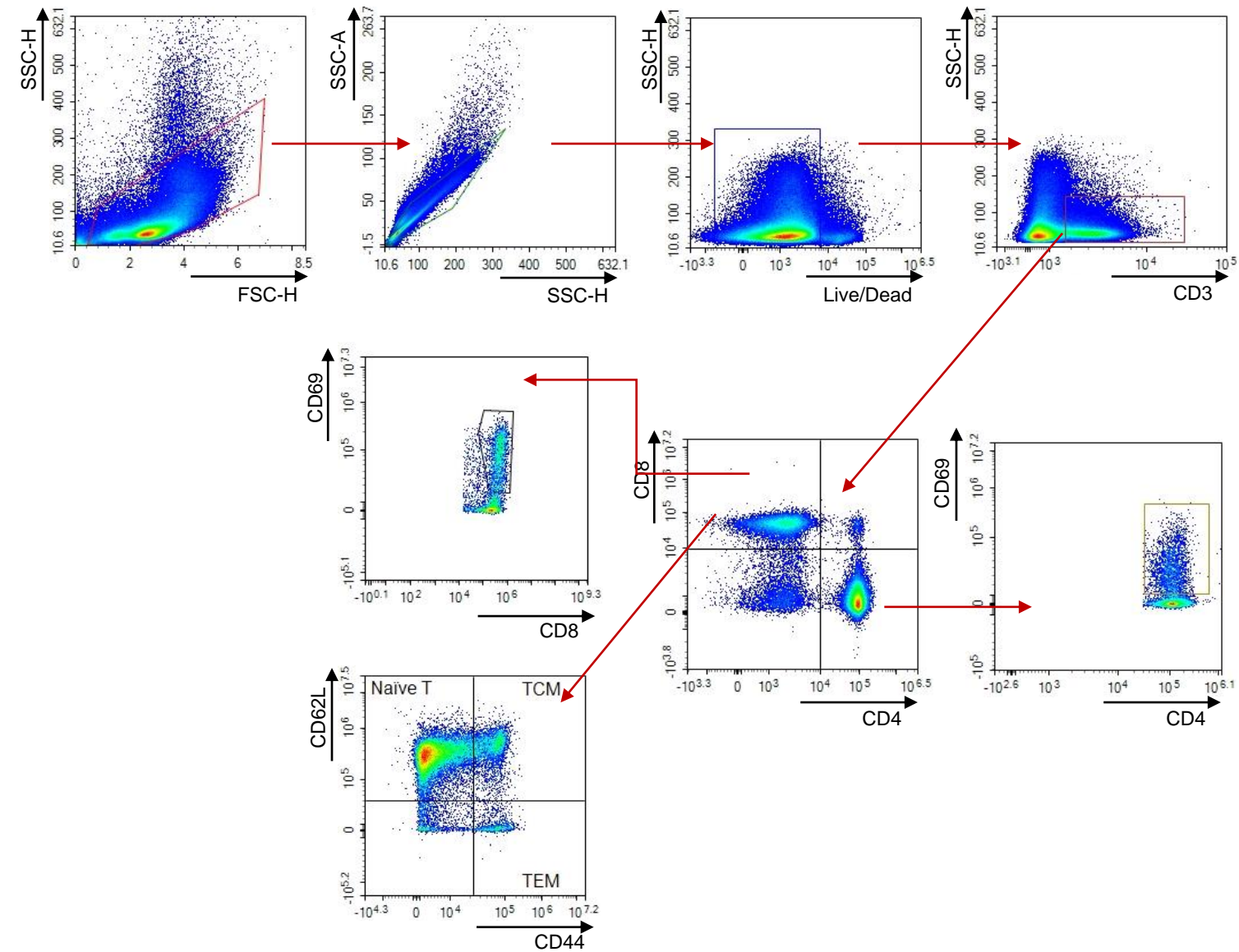

C

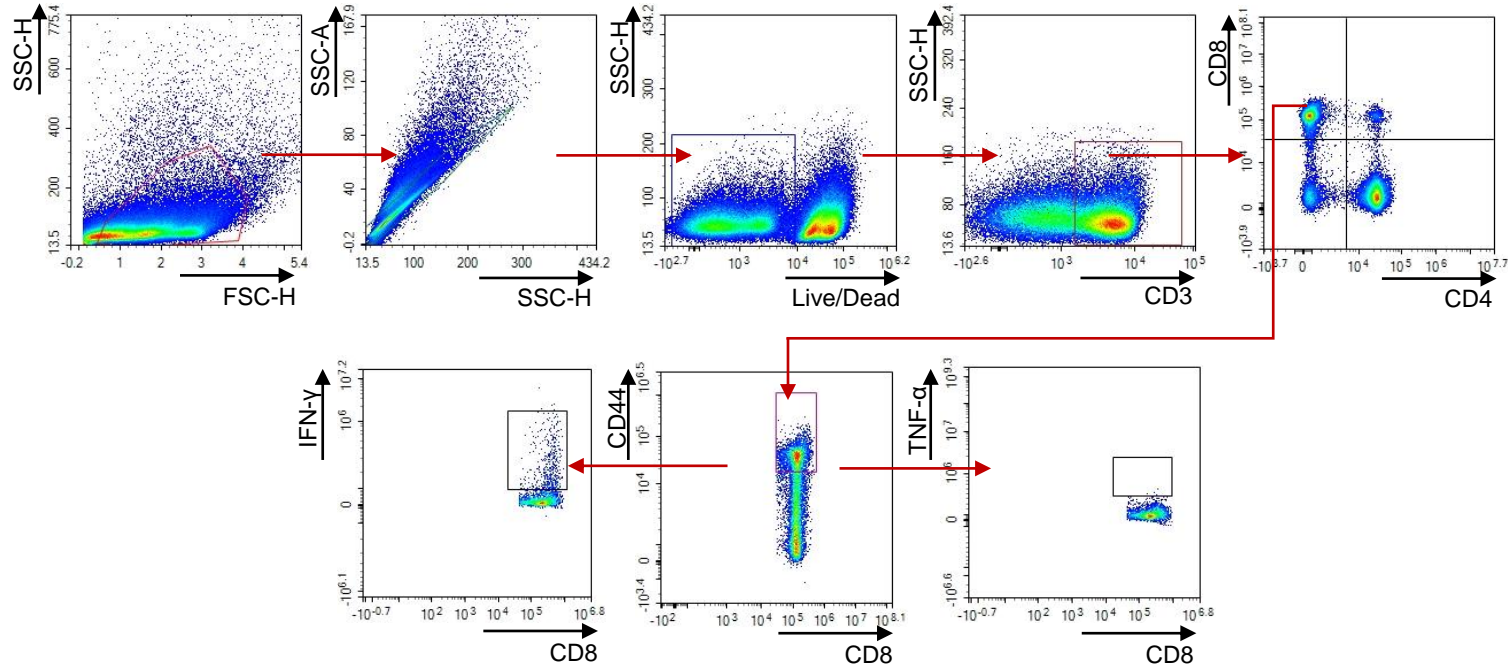

Supplement: Supplementary file 2 — Supporting Information [file CTM2-15-e70305-s001.pdf]

A

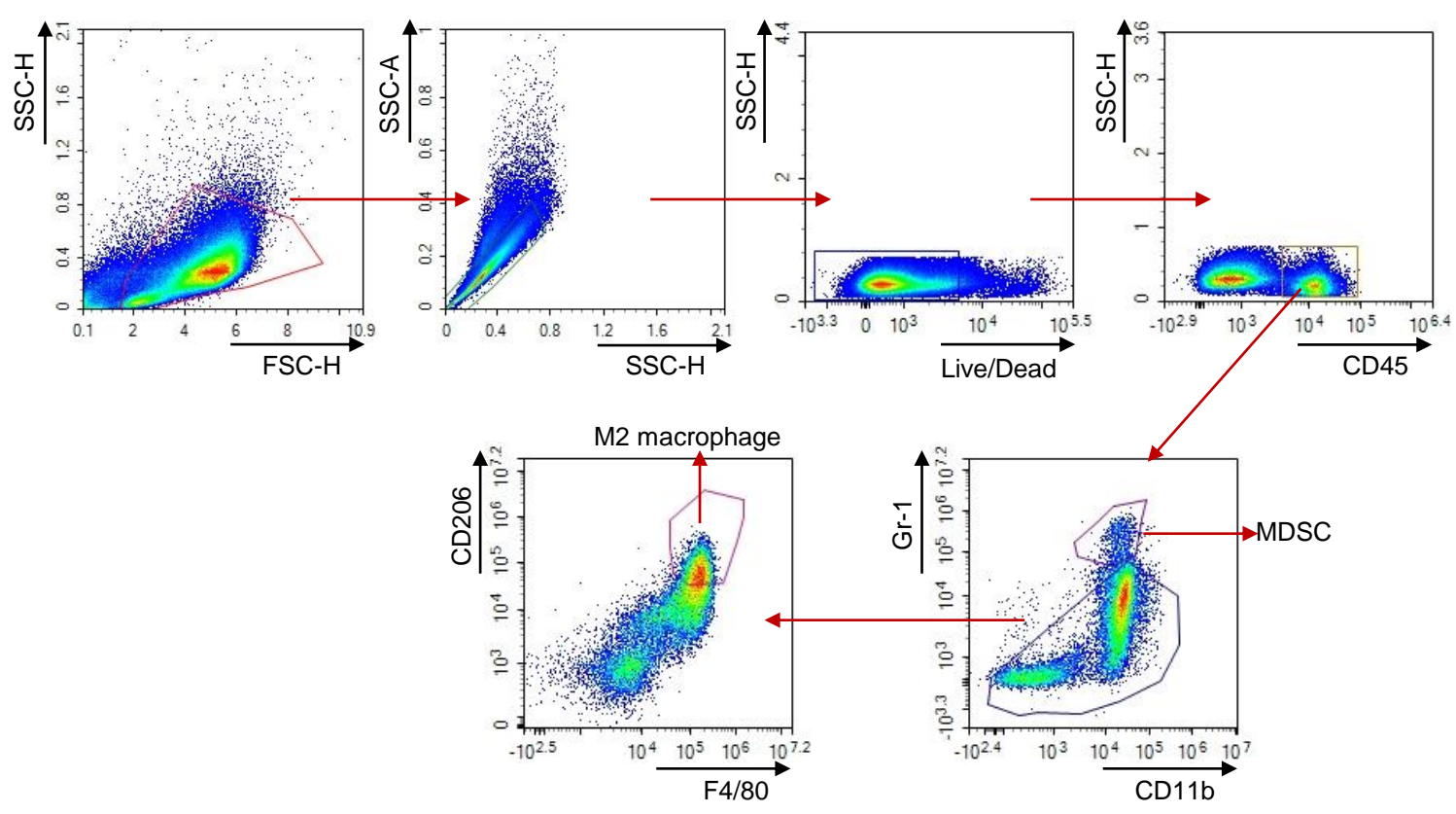

Supplement: Supplementary file 3 — Supporting Information [file CTM2-15-e70305-s003.pdf]

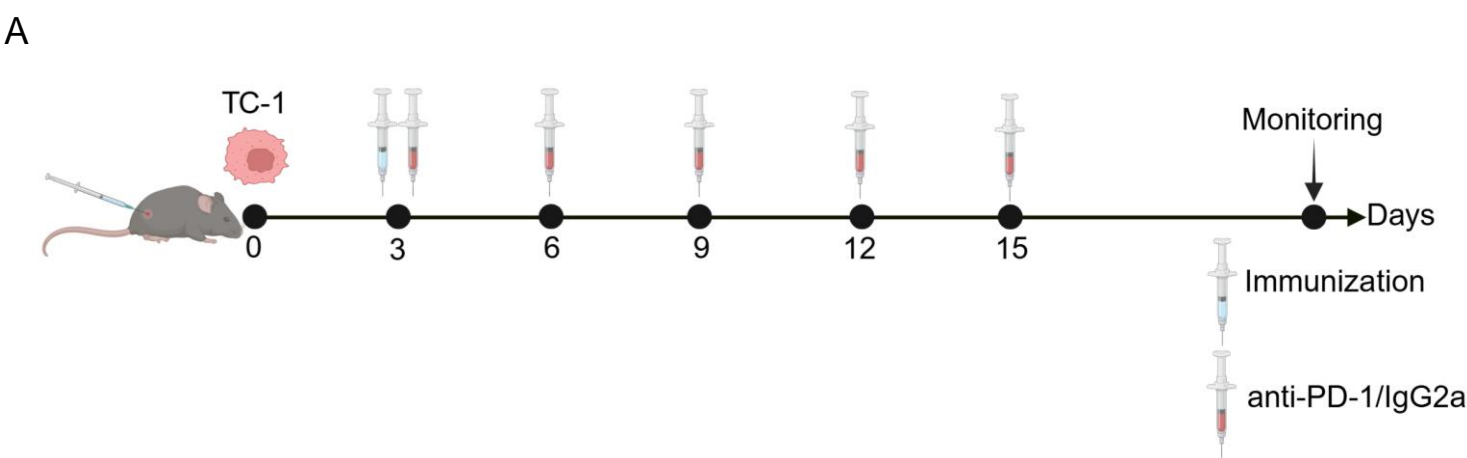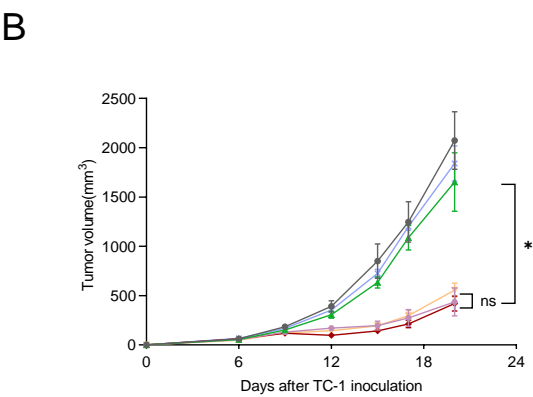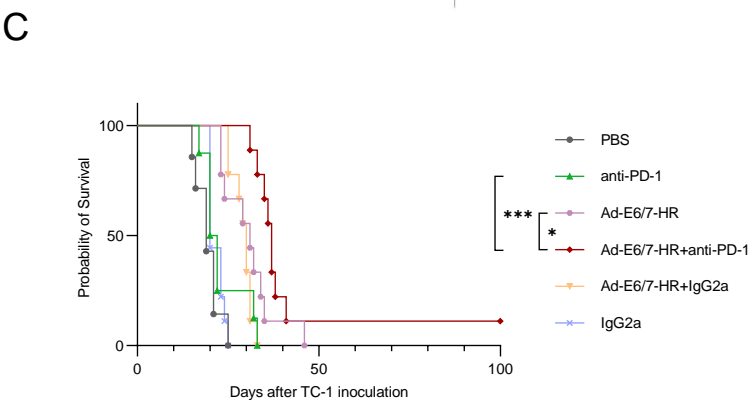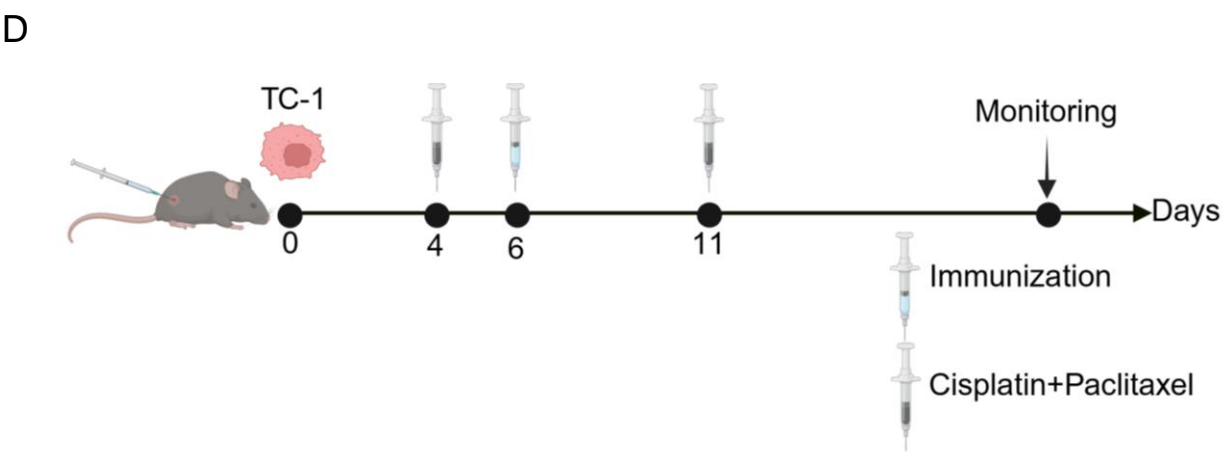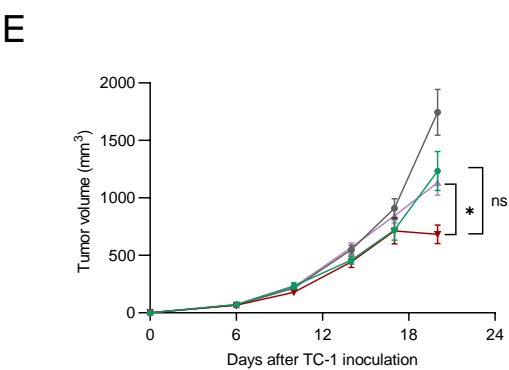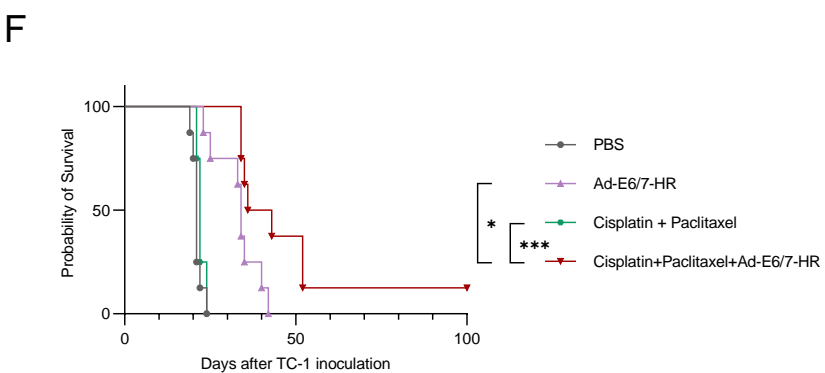

Supplement: Supplementary file 4 — Supporting Information [file CTM2-15-e70305-s002.pdf]

A

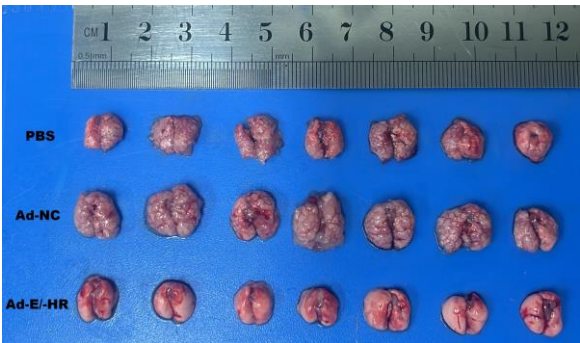

B

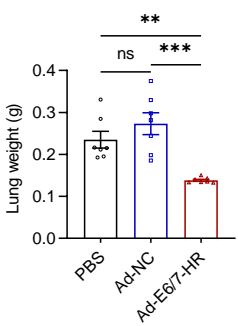

C

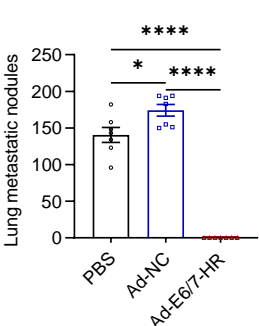

D

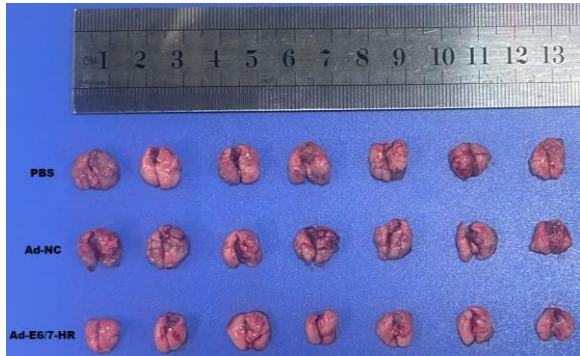

E

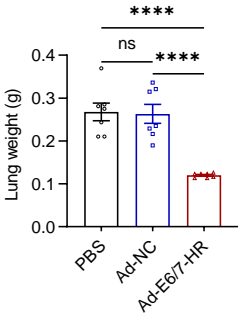

F

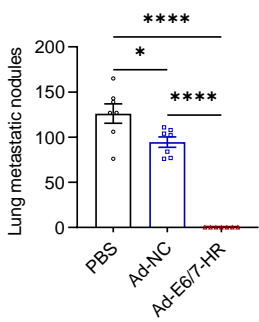

Supplement: Supplementary file 5 — Supporting Information [file CTM2-15-e70305-s005.pdf]

A

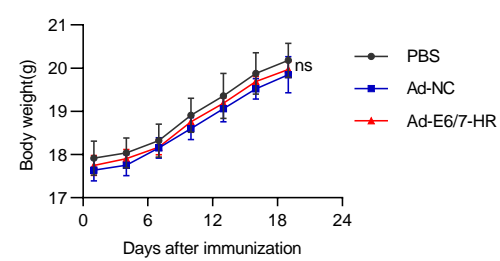

B

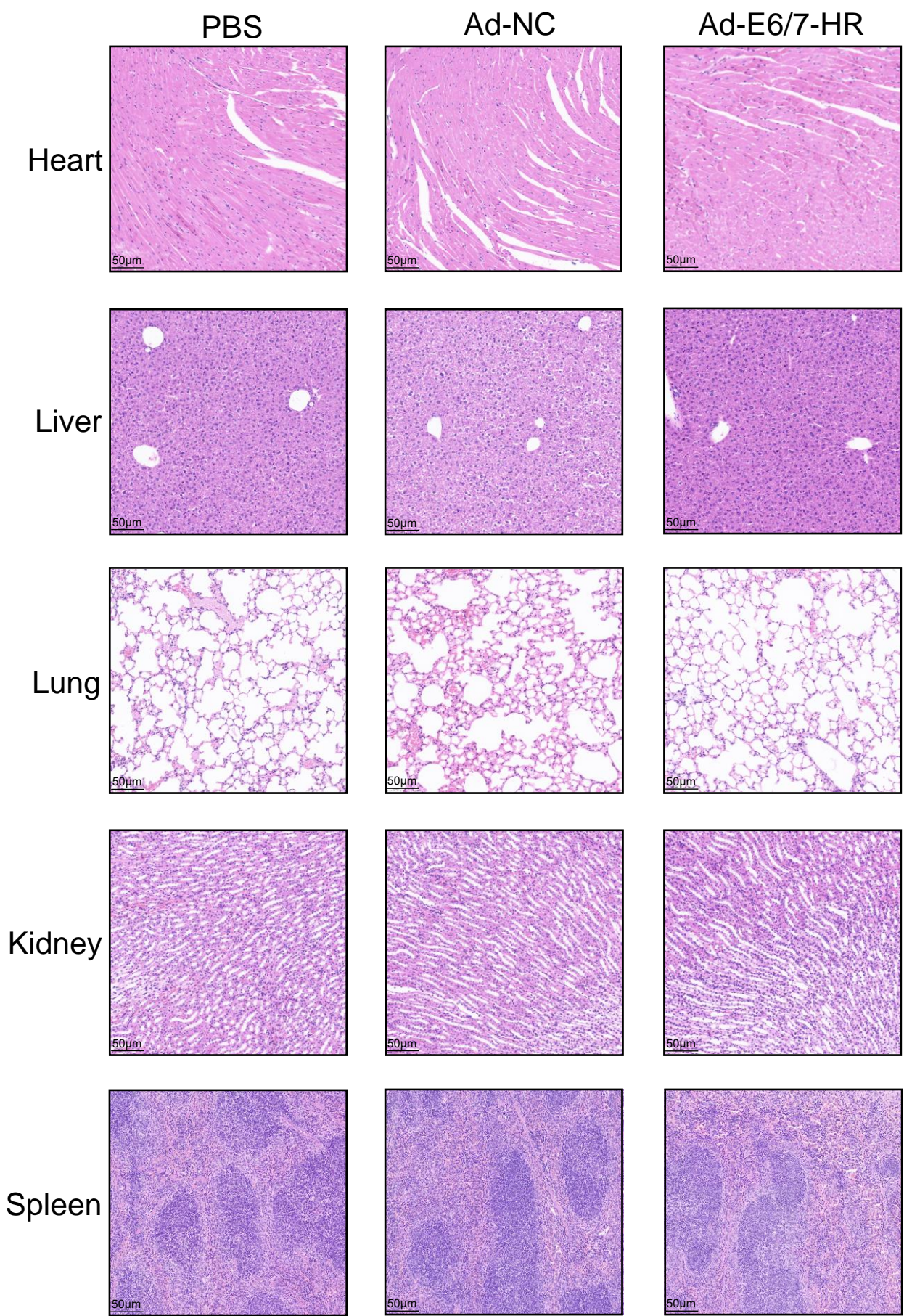

C

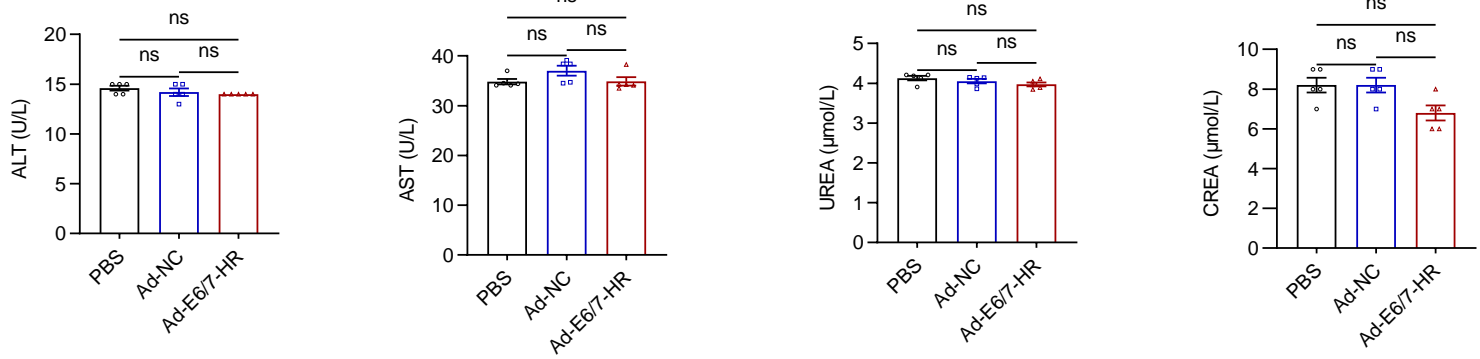

Supplement: Supplementary file 6 — Supporting Information [file CTM2-15-e70305-s004.pdf]
